# A Text-Book of Morbid Histology

**Published:** 1893-03

**Authors:** 


*A Text-Book of Morbid Histology.* By RUBERT BOYCE, M.B., M.R.C.S. Pp. xxiv., 477, with 130 Coloured Illustrations. London: H. K. LEWIS. 1892.

The author treats of hardening, cutting, staining, and mounting sections before describing the various morbid structures. We do not think that the proper way to get good results from the picro-carmine stain is to let the fluid remain on the section only a minute or so; and we are surprised that Farrant's solution has not superseded glycerine as a mounting medium, as it is so much better for the purpose, especially where sections are to be kept for a considerable time.

The study of acute inflammation, the fate of the exudates, repair, chronic inflammation and healing, degeneration, infiltration, pigmentation, infective processes, tumours, cysts, and congenital formations logically precedes that of the special organs; and the author wisely devotes over two hundred pages to these matters. As a whole, this part of the book is concisely and well written, and many of the illustrations are very good, but some of them are capable of much improvement. Especially is this true of lardaceous degeneration of the liver, facing page 84, and of those of myoma and fibroma, facing page 132.

We wish that the author had given us descriptions of the naked-eye appearances of the diseased organs as well as of the microscopic ones, since there is no doubt that valuable pathological structures are often thrown away in the *post-mortem* room from ignorance of naked-eye pathology. The author has performed his task of describing microscopic appearances very well indeed. The book contains a good bibliography, which is valuable for reference to original papers, and the names of foreign writers are mostly correctly given; but why should the name of so well-known an English pathologist as Delépine be wrongly spelled? We hope that this book will be widely read, since the man who builds his medical house on sound pathological knowledge builds it on a rock.

